# Appropriateness of laboratory expenditure for primary health care facilities across South Africa

**DOI:** 10.4102/phcfm.v15i1.3740

**Published:** 2023-06-19

**Authors:** Ozayr Mahomed, Naseem Cassim

**Affiliations:** 1 Department of Public Health Medicine, University of KwaZulu-Natal, Durban, South Africa; 2 Department of Haematology and Molecular Medicine, Faculty of Health Sciences, University of Witwatersrand, Johannesburg, South Africa; 3 National Health Laboratory Service, Johannesburg, South Africa

**Keywords:** ideal clinic, Essential Laboratory List test, primary health care, healthcare expenditure, South Africa, appropriateness, national health insurance

## Abstract

**Background:**

Primary health care (PHC) services have been prioritised from a cost-containment perspective. To manage expenditure, facility managers use the Laboratory Handbook that indicates the Essential Laboratory List (ELL) tests.

**Aim:**

The aim of this study was to analyse PHC laboratory expenditure to assess the impact of the ELL in South Africa.

**Setting:**

We reported ELL compliance at the national, provincial and health district levels.

**Methods:**

A retrospective cross-sectional study was used to analyse data for the 2019 calendar year. The unique tariff code descriptions were used to develop a lookup table to identify ELL compliant testing. Researchers analysed data for the human immunodeficiency virus (HIV) conditional grant tests and by facility for the bottom two districts.

**Results:**

There were 356 497 tests (1.3%) that were not ELL compliant that equated to an expenditure of $2.4 million. Essential Laboratory List compliance ranged from 97.9% to 99.2% for clinics, community healthcare centres and community day centres. The provincial ELL compliance ranged from 97.6% for the Western Cape to 99.9% for the Mpumalanga province. The average cost per ELL test was $7.92. At the district level, ELL compliance ranged from 93.4% for Central Karoo to 100% for Ehlanzeni.

**Conclusions:**

High levels of ELL compliance have been demonstrated from the national to the health district level, demonstrating the value of the ELL.

**Contribution:**

This study provides data for quality improvement initiatives at primary care facilities.

## Introduction

Public health expenditure in South Africa doubled from R107 billion in 2010–2011 to R222bn at the end of the 2019–2020 financial period.^1^ Annual growth in health expenditure averaged around 8.5% between 2012–2013 and 2015–2016; however, the downturn in the economic growth in real terms declined by 1.8% per annum.^1^ Coinciding with the downturn in the economy, the uninsured population (i.e. those without medical aid) has also grown substantially between 2012–2013 and 2019–2020 eroding the per capita real trend.^1^ In order to mitigate for the decreasing budget and expanding demands, the health sector has instituted a number of contingency measures to reduce health expenditure, such as: (1) control of personnel and medicine costs, (2) protection of ‘non-negotiable’ budget items, (3) saving on nonessential items, (4) reduction in capital spending on buildings and (5) the prioritisation of primary health care (PHC) and chronic medicine dispensing and the distribution at additional and alternate sites to reduce queues and improve access with a focus on health outcomes.^1^ Of particular relevance to the focus of the current research is that the average growth in expenditure for laboratory services was 3.4% between 2012–2013 and 2019–2020, which is above the real growth in the budget.^1^

**FIGURE 1:**
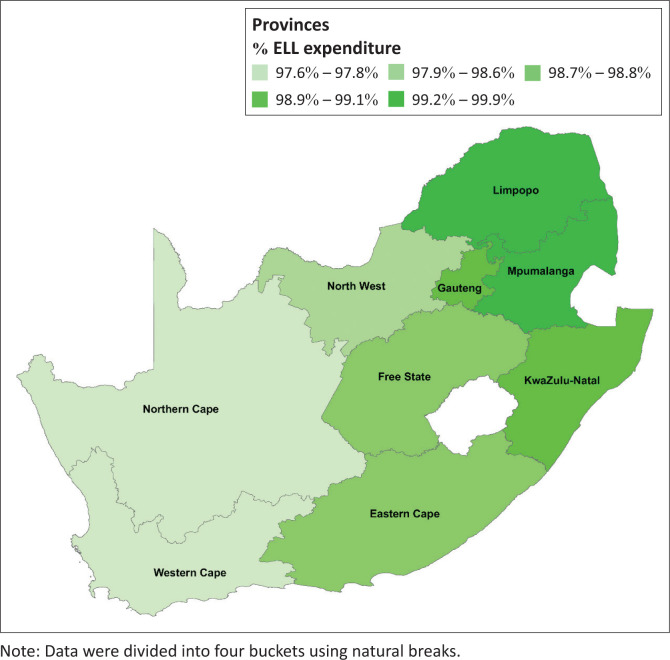
Choropleth map showing the percentage of provincial laboratory expenditure that is compliant with the Essential Laboratory List test for primary health care services in 2019, South Africa.

Primary health care services have been prioritised both from a cost-containment perspective and improving health outcomes of the population by improving the quality of healthcare services. The Ideal Clinic Realisation and Maintenance (ICRM) programme aims to improve the effectiveness and responsiveness of the health system specifically at the PHC level, which was launched by the Director General of the National Department of Health (NDOH) in 2013.^2^ The ICRM programme provides the minimum inputs and processes required from PHC facilities to deliver a desired output (Figure 1). An Ideal Clinic is defined as:

[*A*] clinic with good infrastructure, adequate staff, adequate medicine and supplies, good administrative processes and sufficient bulk supplies, that uses applicable clinical policies, protocols, guidelines as well as partner and stakeholder support, to ensure the provision of quality health services to the community.^3^ (p.4)

The ICRM framework consists of 10 components as follows: (1) administration, (2) integrated chronic disease and/or clinical services management, (3) medicines, supplies and laboratory services, (4) human resources for health, (5) support services, (6) infrastructure, (7) health information management systems, (8) communication, (9) district health system support and (10) implementing partners and stakeholders. Each of these components have one or more subcomponents. Overall, there are 10 components and 32 subcomponents.

Component three and subcomponent 13 refer to the management of laboratory services. Subcomponent 13 defines a number of aspects the facility manager is responsible for as follows: (1) PHC laboratory handbook is available; (2) required functional diagnostic equipment and concurrent consumables for point-of-care testing are available; (3) required specimen collection materials and stationery are available; (4) specimens are collected, packaged, stored and prepared for transportation according to the PHC laboratory handbook; (5) laboratory results are received from the laboratory within the specified turnaround times; (6) facility is enrolled as a testing point in the National Health Laboratory Service (NHLS) human immunodeficiency virus (HIV) proficiency testing scheme and (7) facility controls rapid test kit performances by running one negative and one positive control on a weekly basis.^3^ To manage laboratory expenditure, facility managers are required to use the PHC Laboratory Handbook that indicates the Essential Laboratory List (ELL) tests.^4^ Furthermore, a dedicated laboratory request form has been introduced based on the ELL. The PHC facility manager is now responsible for developing control measures for the rational utilisation of laboratory expenditure in line with evidence-based guidelines.^5^

Laboratory demand management aims to improve the requisition of the appropriate laboratory test as well as result in the reductions in public health expenditures without affecting clinical outcomes.^6^ It is incumbent for the health authority to develop a broad-based demand management strategy that defines what is appropriate test based on the clinical services offered.^6^ This may involve standardising the repertoire of tests that may be requested by level of care.^6^ A local study stated that laboratory services will have to formulate strategies to address both under- and overutilisation of laboratory tests and ensure that the proper use of clinical laboratory testing contributes to improved patient care.^7^ This will require laboratories to monitor test usage for cost-effectiveness and appropriateness.^7^ Some studies have used rule-based systems or algorithms to implement laboratory demand management that is more suited to higher levels of care.^8,9,10^ Other laboratory demand management strategies include education, the redesign of laboratory request forms, use of computerised physician order entry (CPOE) and implanting reimbursement models.^11^ In a South African setting, the development of an ELL list that is implemented using a standardised PHC laboratory request appeared to be the most optimal demand management strategy.

The NHLS operates a platform of over 268 diagnostic laboratories across South Africa serving 80% of the population.^12^ The NHLS operates a platform of 226 laboratories across South Africa, ranging from highly sophisticated centralised, high volume and academic to low test volume distant rural laboratories.^12^ The core function of the NHLS is to provide cost-effective and efficient health laboratory services in the public healthcare sector.^12^ A previous retrospective cross-sectional analysis of laboratory expenditure for the 2013–2014 financial period for 11 pilot National Health Insurance (NHI) health districts reported that approximately R35 million South African Rand (10%) of the estimated R339m expenditures was not ELL compliant, that is, tests not listed in the ELL.^13^ This indicates that up to 13% of testing conducted at PHC clinics was not ELL compliant at the time.^13^

### Aims and objectives

This study aims to analyse the laboratory expenditure across 52 health districts to assess ELL compliance. A secondary aim was to assess whether the ideal clinic initiatives introduced have resulted in the widespread adoption of the ELL across South Africa.

## Research methods and design

### Study design

A retrospective cost analysis was conducted for laboratory expenditure data for PHC services across South Africa for the 2019 calendar year.

### Study setting and population

Data were extracted for all PHC health facilities across 52 health districts. We used the district health information system (DHIS) organisational hierarchy to include expenditure for PHC clinics, community health centres (CHCs) and community day centres (CDCs). No sampling was performed as all PHC facilities with laboratory expenditure data were included. A comprehensive list of account numbers was obtained from the NHLS Finance department. We used this list to identify account numbers associated with PHC facilities.

### Data collection and preparation

We received three separate data extracts: (1) PHC billing accounts expenditure data for all PHC account numbers – routine tests requested on the PHC request form (N1) and allocated to the default billable account number (e.g. 32ZKOK000005 for the Kokosi Clinic), (2) Xpert MTB/RIF conditional grant expenditure identified by the prefix ‘ZGXP’ and (3) comprehensive care, management and treatment (CCMT) of HIV programme conditional grant expenditure identified by the prefix antiretorviral expenditure (ZARV). Data were received as a password-protected file and personally collected from the corporate data warehouse (CDW).

Each data extract included the following variables: (1) billable account number (e.g. 16ZJEF000001 for PHC facilities in the Kouga municipality), (2) customer name, (3) facility code, (4) facility description, (5) year, (6) month, (7) tariff code, (8) tariff description, (9) test volumes and (10) expenditure. The expenditure for a laboratory test is itemised as tariff codes; for example, tariff code 2210 denotes the haemoglobin test.^13^

The tariff code and tariff description are assigned within the laboratory information system (LIS) for each test. There is both a one-to-one and one-to-many relation between a tariff code and a test. For example, the alanine transaminase (ALT) test has a single tariff code (2685) when compared with the lipogram panel, which has multiple tariff codes for each analyte, for example, total cholesterol (2855), triglyceride (3440), high-density lipoprotein (HDL) (2865) and the calculated low-density lipoprotein (2860). A panel is a series of tests performed on one specimen that is usually related to a single condition or disease.^14^ Each tariff code is associated with a state price that determines expenditure, that is, test volume multiplied by the tariff code state price.

SAS 9.4 (Cary, NC, USA) was used to append these three expenditure datasets, ensuring that variables were in the same data type format (number or string) with the same header row. The appended SAS dataset was used to commence with data preparation.

The authors used the tariff codes to identify ELL compliant testing (test listed in the ELL). We extracted all unique tariff codes reported in the appended dataset and developed a lookup table to assign the following values with examples provided in brackets: (1) ELL status (ELL Test or Not an ELL Test); (2) test description (cholesterol); (3) discipline (chemical pathology) and (4) test group (lipogram). This was performed by a team of experts involved in the Ideal Clinic Initiative. However, as PHC clinical guidelines have changed since the ELL was first published in 2018, the researchers have amended the lookup table to include additional tests such as alpha-fetoprotein (AFP) introduced for neural tube defect screening.^4^ Each test added to the ELL lookup table was based on the defined package of services offered by PHC services.^15^

The lookup table approach was used as it allows the physical representation of attributes, that is, variables in a relational database.^16^ They can be used to store information for one or more related attributes.^16^ The lookup table aimed to identify laboratory expenditure that was ELL compliant.

For each tariff code, the ELL status, test description, discipline and test group variables were coded by a team of experts. For tests not in the ELL, data were categorised as ‘Not an ELL’ test compared with ‘ELL test’. The test group identified panels where more than one tariff code was reported for a test (e.g. lipogram).

All unique facility descriptions were extracted and matched to the DHIS organisation hierarchy. For each facility, the following DHIS variables were added: (1) Organisational Unit (OU) 5 (facility name), (2) OU3 (health district), (3) OU2 (province) and (4) OU_Type (facility type).

A structured query language (SQL) procedure was used to create a left outer join between the appended laboratory expenditure dataset, tariff code and facility lookup tables, with the tariff code and facility description as primary keys. All non-PHC expenditure data was excluded using the DHIS OU_Type variable, that is, researchers included clinics, CHC and CDC. This resulted in the creation of a new SAS dataset for PHC laboratory expenditure indicating ELL compliance for each row of data.

A series of data quality checks were conducted before commencing with the data analysis. These included: (1) ensuring that all tariff codes had an ELL status populated, (2) identifying that the top 10 tariff codes based on expenditure match national NHLS volume and revenue reports (data not shown), (3) ensuring that no expenditure data were reported for hospitals and (4) checking that data were reported for all nine provinces and 52 health districts. All laboratory expenditure data were obtained in South African Rands (ZAR) and converted to United States dollars (USD) using an exchange rate of 14.450 (period average for the year).^17^

### Statistical analysis

The data analysis was conducted using SAS 9.4 and Stata SE (Stata Corporation, College Station, TX, USA). At the national level, researchers reported test volumes and the percentage of laboratory expenditure that was ELL compliant. This data was compared with the overall NHLS test volumes and expenditures for the 2019–2020 financial period. The percentage of test volumes and laboratory expenditure that was ELL compliant by facility type was also reported on. ArcGIS from ESRI (Redlands, CA, USA) was used to create choropleth maps of the percentage of ELL compliance at the provincial and health district levels, using four buckets assigned using natural breaks (Jenks). This is a data classification method that assigns values into classes (e.g. intervals of 10). The shapefiles were obtained from the Municipal Demarcation Board.^12^ In addition, laboratory expenditure and test volumes data were used to calculate the provincial average cost per test for appropriate (an ELL test) and inappropriate (not an ELL test) expenditure. The researchers assessed what percentage of ELL compliant expenditure was related to core HIV conditional grant testing (HIV viral load, CD4 and HIV DNA PCR), with the remaining tests categorised as Other. For the two health districts with the lowest percentage of ELL compliance, researchers reported a histogram of ELL compliance for each health facility (which were anonymised). The DHIS coordinates was also used to show the percentage of ELL compliance for each health facility (using graduated symbols). The map was used to identify any clusters of poor ELL compliance.

### Ethical considerations

Ethical approval was obtained from the Human Research and Ethics Committee (HREC) (Medical) at the University of Witwatersrand (M200545). Approval to extract expenditure data was obtained from the NHLS Academic Affairs, Research and Quality Assurance (AARQA) Department. The expenditure data were extracted by the CDW, which is the national repository of all public section laboratory data generated by NHLS laboratories. No patient identifiers were collected and therefore informed consent was not required.

## Results

Laboratory expenditure data are presented for 3354 PHC facilities. There were 3031, 66 and 257 clinics, CDC and CHC, respectively. Of the total PHC expenditure, 25.6%, 7.6% and 66.9% were for PHC billing accounts, Xpert MTB/RIF and CCMT conditional grants, respectively.

### National laboratory expenditure analysis

At the national level, 98.9% and 98.7% of expenditure and test volumes were ELL compliant (Table 1). There were 356 497 tests (1.3%) that were not ELL compliant that equated to an expenditure of $2.4m. Overall, PHC services represented 38.5% of laboratory expenditure and 29.3% of test volumes when compared with all public sector data reported by the NHLS for the 2019–2020 financial period.

**TABLE 1:** National laboratory expenditure and test volumes for the 2019 calendar year by Essential Laboratory List (ELL) test status for primary healthcare (PHC) facilities in South Africa.

ELL status	Laboratory expenditure (USD)	Test volumes
ELL test	$216 124 904 (98.9%)	27 274 945 (98.7%)
Not an ELL test	$2 395 653 (1.1%)	356 497 (1.3%)
**Total**	**$218 520 557**	**27 631 442**
2019/2020 NHLS^a^	$567 422 234	94 209 082
% PHC	38.5%	29.3%

NHLS, National Health Laboratory Service; USD, United States dollars.

a Public sector laboratory expenditure data for all levels of care (NHLS).

Note: Data are reported in USD.

### Analysis by facility type

The majority of expenditure was for clinics (75.8%). In comparison, 19.5% of expenditure was for CHC. Community day centres are predominantly available in the Western Cape and accounted for 4.7% of expenditure. The percentage of laboratory expenditure that was ELL compliant was 99.2%, 98.1% and 97.9% for clinics, CHC and CDC, respectively (Table 2).

**TABLE 2:** National laboratory expenditure analysis by Essential Laboratory List test status by facility type as defined in the District Health Information System for primary health care services in 2019, South Africa.

Facility type	ELL test	Not an ELL test	Total	ELL compliant (%)
Clinic	$164 325 868	$1 373 376	$165 699 244	99.2
Community health centre	$41 749 299	$810 949	$42 560 248	98.1
Community day centre	$10 049 737	$211 328	$10 261 065	97.9

Note: Data are reported in United States dollars (USD).

ELL, essential laboratory list.

### Provincial laboratory expenditure analysis

**FIGURE 2:**
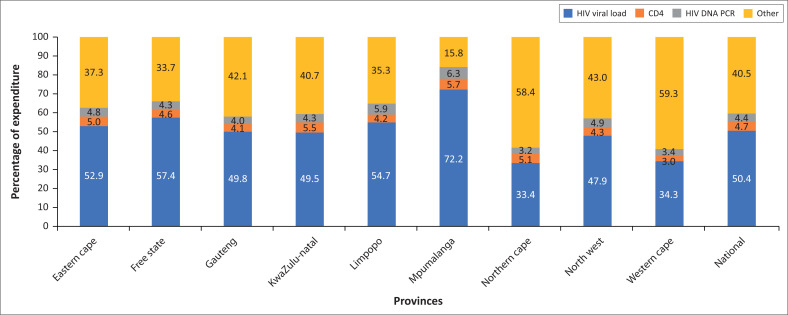
Bar chart showing the provincial breakdown on primary health care Essential Laboratory List test expenditure for the three HIV conditional grant tests (HIV viral load, CD4 and HIV DNA PCR) with the remaining tests classified as Other for primary health care facilities in 2019, South Africa.

Mpumalanga province was the most compliant in terms of reported ELL complaint expenditure. The provincial percentage of ELL compliant expenditure ranged from 97.6% for the Western Cape to 99.9% for the Mpumalanga province. Two provinces reported ELL complaint expenditure between 99.2% and 99.9% (Limpopo and Mpumalanga). The Gauteng and KwaZulu-Natal provinces reported percentages between 98.9% and 99.1% (Figure 2). Three provinces reported percentages between 98.7% and 98.6%. Only the Western and Northern Cape provinces reported an ELL compliant percentage ≤ 97.8%.

The detailed national expenditure analysis revealed an average cost per test of $7.92 and $8.17 for an ELL and non-ELL testing, respectively. For ELL compliant testing, the average cost per test ranged from $6.51 to $12.40 for the Northern Cape and Mpumalanga provinces, respectively. The average cost for non-ELL testing ranged from $6.11 (Western Cape) to $8.23 (Gauteng) (Table 3).

**TABLE 3:** Provincial laboratory expenditure and test volumes analysis to determine the average cost per test for non–Essential Laboratory List testing by primary healthcare services in 2019, South Africa.

Province	ELL test			Not an ELL test		
	Laboratory expenditure (USD)	Test volumes	Average cost per test (USD)	Laboratory expenditure (USD)	Test volumes	Average cost per test (USD)
Eastern Cape	$22 628 713	2 952 655	$7.66	$309 056	45 873	$6.74
Free State	$11 155 941	1 198 539	$9.31	$138 909	20 704	$6.71
Gauteng	$53 217 440	6 936 658	$7.67	$493 835	60 020	$8.23
KwaZulu-Natal	$61 584 048	7 564 959	$8.14	$592 144	93 741	$6.32
Limpopo	$14 365 383	1 908 709	$7.53	$60 169	9314	$6.46
Mpumalanga	$14 840 932	1 197 025	$12.40	$10 675	1402	$7.61
Northern Cape	$4 561 965	700 693	$6.51	$101 545	15 559	$6.53
North West	$14 122 386	1 817 078	$7.77	$196 711	29 296	$6.71
Western Cape	$19 648 096	2 998 629	$6.55	$492 610	80 588	$6.11

**National**	**$216 124 904**	**27 274 945**	**$7.92**	**$2 395 653**	**356 497**	**$6.72**

ELL, Essential Laboratory List; USD, United States dollars.

Note: Data were reported in USD.

The analysis of the HIV conditional grant expenditure revealed that the HIV viral load test contributed 50.4% of expenditure nationally. At the provincial level, this ranged from 33.4% to 72.2% for the Northern Cape and Mpumalanga provinces, respectively. For CD4 and HIV DNA PCR testing, a range of 3.0% – 5.7% and 3.2% – 6.3% was reported, respectively. The other tests category was lowest for the Mpumalanga province (15.8%) compared with a national value of 40.5%.

### Health district laboratory expenditure analysis

**FIGURE 3:**
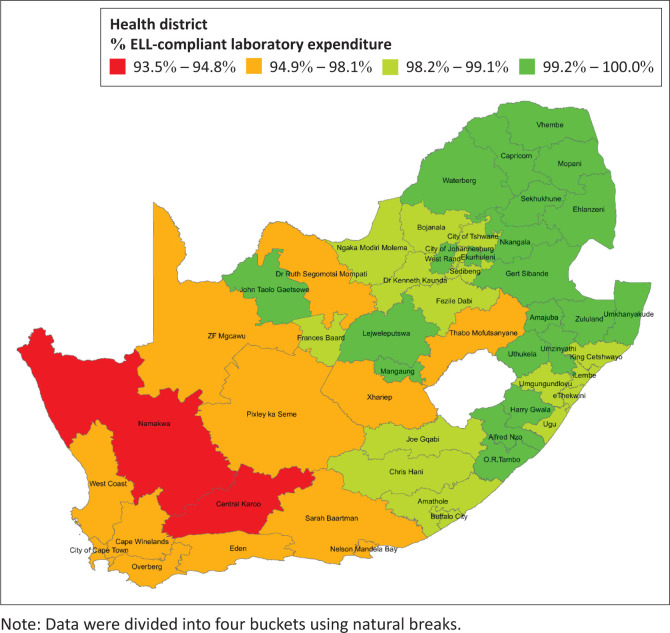
Choropleth map showing the percentage of health district laboratory expenditure that is compliant with the Essential Laboratory List (ELL) test for primary healthcare (PHC) services in 2019, South Africa.

At the health district level, ELL compliant expenditure ranged from 93.4% for the Central Karoo (Western Cape) to 100% for Ehlanzeni (Mpumalanga). A range of 99.2% – 100% was reported for 19 of the 52 districts (36.5%). Furthermore, 15 of the 52 (28.8%) districts reported a percentage of ELL compliant expenditure between 98.2% and 99.1% (Figure 3). Only two districts reported ELL compliant laboratory expenditure of less than 94.8% (Namakwa and Central Karoo).

### Health facility analysis for the two worst health districts (Namakwa and Central Karoo)

Across the 28 facilities in the Namakwa District, the median compliance rate to ELL was 95.6% (interquartile range [IQR]: 92.7% – 97%). The lowest compliance was 89.7% from a single facility. Twenty-five percent (*n* = 7) of facilities reported an ELL compliance rate of ≤ 93%. There was no statistical difference in the ELL compliance rate between these two districts (*p* > 0.05). Across the 10 facilities in Central Karoo District, the median ELL compliance rate was 93.5% (IQR: 92.7% – 94.3%). The lowest compliance was 90.9% at a single facility.

**FIGURE 4:**
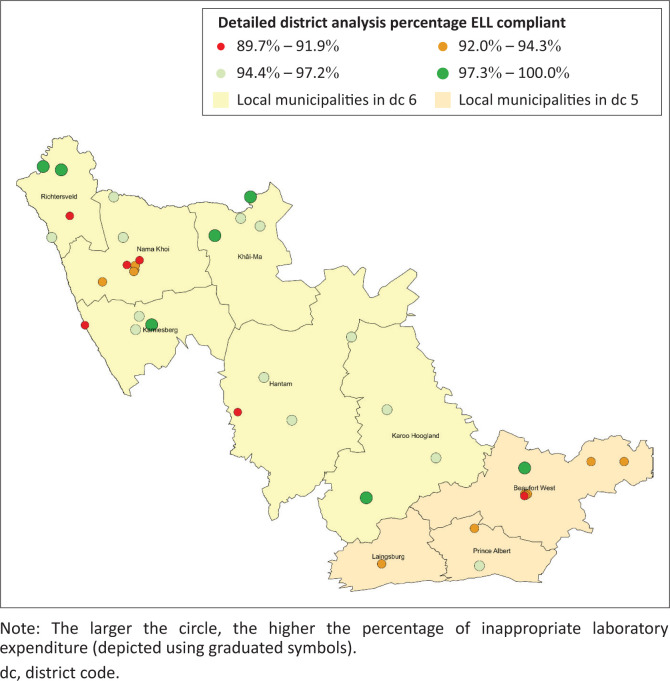
Map showing the percentage of primary health care laboratory expenditure that was Essential Laboratory List test compliant in the 2019 calendar year for each health facility for the two worst districts (Namakwa and Central Karoo).

The spatial analysis at the health facility level in the Namakwa and Central Karoo health districts revealed no clusters of poor ELL compliance (Figure 4). There were five health facilities with ELL compliance between 89.7% and 91.9% (red circles).

## Discussion

The objective of this study was to assess compliance of laboratory expenditure across 52 health districts to a PHC ELL in South Africa. Primary health care services have been exposed to the ideal clinic initiative that aims to improve the utilisation of minimum inputs and processes to deliver a desired health output.^5^ The development of an ELL is a demand management approach that achieves cost containment, standardisation of practices and better health outcomes for the population by improving the quality of healthcare services.^5^

The high level of ELL compliant laboratory expenditure (> 98%) is a promising finding and is indicative of the successful implementation of a combination of demand management interventions. Furthermore, the finding from this study is an improvement from the previous 90% reported for the NHI pilot districts.^13^ This improvement indicates that these interventions have continued to yield good levels of ELL compliance many years later. The combination of the ELL and a standardised laboratory request form have made it possible to extend work for these pilot districts to all PHC across South Africa to around 3800 facilities.

This indicates that these interventions have been implemented across PHC facilities with similar levels of ELL compliance despite the variation in operating hours, package of services offered, size of the facility and the percentage of healthcare offered by doctors between clinics and community healthcare centres. Furthermore, across nine provinces, ELL compliance was over 97%. This is a positive finding that indicates that the interventions have been cascaded across all PHC facilities in South Africa and is indicative of the sustainable intervention of laboratory expenditure demand management. It should be noticed that Free State, Limpopo, Mpumalanga, Northern Cape, has good compliance probably because of low testing carried out on the population.

In developing countries, patient records are collected on pen and paper systems used by clinical staff and then entered later into an information system by data entry staff either locally or remotely.^18^ The absence of a medical record system specifically for PHC services makes it difficult to develop more advanced methods of demand management such as rule-based systems and/or algorithms.^8,9,10^ Even with the ideal clinic system, aggregate reporting is based on the use of standardised clinical stationery and registers that collate data using systems such as Tier.Net, DHIS and Health Patient Registration System (HPRS).^19,20,21^ In the absence of a more advanced data system, it would be difficult to implement more advanced demand management strategies. There have been demand management approaches deployed at hospitals in South Africa where rule-based algorithms have been deployed on the LIS.^10,22,23,24^ Some of these approaches could be deployed to PHC services. However, it must be observed that a local study reported that these approaches do not appear to be effective with limited impact on clinician test requesting pattern.^24^ In addition, with such high levels of ELL compliance, the rule-based algorithm would have a much smaller impact at PHC facilities because of the presence of a limited test repertoire.

The provincial ELL compliance ranged from 97.6% (Western Cape) to 99.9% (Mpumalanga province). Furthermore, the ELL compliance was over 93% for 52 districts, ranging from 93.4% (Central Karoo, Western Cape) to 100% (Ehlanzeni, Mpumalanga). This confirms that ELL compliance is widespread and has been expanded to all PHC facilities across South Africa. The bottom two districts reported ELL compliance rates of 93.4% (Central Karoo) and 95.6% (Namakwa). These are two adjoining rural districts that are situated in the south western part of South Africa with a population of 472 603 and 116 205, respectively, in 2022.^25^ The analysis at the health facility level revealed that there were five health facilities with ELL compliance ≤ 91.9% in these two districts. This indicates that poor performance at a few facilities impacted the performance of these two districts. This indicated that targeted interventions at poor-performing health facilities would be more appropriate.

The analysis of the provincial average test cost for ELL compliant testing revealed a wide range from $6.55 (Western Cape) to $12.40 (Mpumalanga) compared with a national value of $7.42. In particular, the Mpumalanga province reported an average test cost of $4.48 higher than the national value. The analysis of HIV conditional grant testing for the Mpumalanga province revealed that HIV viral load (VL) testing contributed over 70% of expenditure, which is 20% higher than the national value. When we looked at the National Institute for Communicable Disease (NICD) HIV M&E dashboard data for Q4 2019, the percentage of people in care who have a VL performed in the last 12 months (VL coverage) was 75.3% (data not shown) in this province.^26^ This ranged from 54.6% to 92.2% for the Eastern Cape and Western Cape provinces, respectively.^26^ The Mpumalanga province reported a VL coverage of 74.0%.^26^ This finding contradicts the 72.2% contribution of HIV viral load testing to expenditure in this province. The 2017 National Antenatal Sentinel HIV Survey in South Africa reported that the overall HIV prevalence was highest in KwaZulu-Natal (41.1%) followed by the Mpumalanga province (37.3%).^27^ Despite reporting similar HIV prevalence, less than 50% of ELL compliant expenditure was for HIV viral load testing in the KwaZulu-Natal province. This indicates that a higher HIV burden is not able to explain the higher average test cost in the Mpumalanga province. Perhaps, antiretroviral treatment (ART) enrolment data could explain the increased average test cost noticed.

The approaches reported in this study should be developed as an interactive dashboard to make it possible for district managers to quickly flag poor ELL compliance. Similar approaches have demonstrated the value of interactive dashboards to improve turn-around-time (TAT) performance.^28,29^ In addition, HIV and TB cohort monitoring and evaluation dashboards have been used to report key indicators from the national to the health facility level (e.g. virological suppression).^30,31,32^ For the ELL dashboard development, the updated lookup table should be used as it incorporated recent evidence-based recommendations to increase the test repertoire for PHC services. This would make it possible for all levels of healthcare management to assess compliant laboratory expenditure.

### Limitations

This study only assessed ELL compliance. Without the clinical data, it is not possible to determine the appropriateness of the diagnostic testing.

## Conclusion

High levels of ELL compliance have been demonstrated from the national to the health district level. Similarly, across the three categories of PHC services, we observed high levels of ELL compliance. Our findings have demonstrated the value of a national ELL-aligned standardised laboratory request form for PHC services. We identified the need for better monitoring for poor-performing facilities. One of the next steps would be to determine a benchmark percentage for ELL compliance, which would make it easier to identify outliers. In addition, this would make it possible to create routine exception reports for district managers. There is a need to develop an integrated dashboard to rapidly identify poor-performing facilities for targeted interventions and quality improvement programmes.
